# Teachers’ interpersonal styles and fear of failure from the perspective of physical education students

**DOI:** 10.1371/journal.pone.0235011

**Published:** 2020-06-24

**Authors:** Elisa Huéscar Hernández, Juan Antonio Moreno-Murcia, José Espín

**Affiliations:** 1 Health Psychology Department, Miguel Hernández University, Elche, Spain; 2 Sport Research Center, Miguel Hernández University, Elche, Spain; Kyoto University, JAPAN

## Abstract

A growing body of research-based knowledge has been generated for the purpose of better understanding the reciprocal and dynamic relationship between teachers’ instructional characteristics and students’ psychosocial and learning outcomes. This study specifically examined the relationship between teachers’ interpersonal styles and fear of failure outcomes in physical education students. Five hundred sixty-two middle school and high school students in Physical Education classes (PE) participated in the study. Students completed questionnaires that assessed instructors’ autonomy-supportive and controlling teaching styles and students’ own fear of failure. A person-centered analysis was used to test the hypotheses. The results revealed that higher teacher autonomy support was associated with lower student fear of failure. To the contrary, a controlling teaching style was associated with fear of failure in these students. Two profiles emerged in which moderate fear of failure was associated with a stronger perception of a controlling teacher style and lower levels of fear of failure were associated with greater perceived instructor support for autonomy.

## Introduction

Typical physical education classroom learning environments can take on a competitive orientation given the nature of the subject matter and the opportunities available for students to demonstrate their motor skills and physical competence in this publicly visible setting. In this context, it is common for students to express concerns about the extent to which they are expected to attain normative levels of performance and the extent to which their skill characteristics can be evaluated by their peers [1,2]. These types of circumstances can contribute to feelings of pressure, insecurity, anxiety and stress for those students who perceive themselves to be less capable than others as a consequence of previous negative experiences that may have included feelings of embarrassment [3].

Fear of failure has been defined as the tendency to anticipate embarrassment under circumstances of failure [4,5]. Although there seems to be a consensus that occasional failure is necessary to make subsequent progress in most contexts, in the academic context failure can be problematic [6]. Fear of failure is more likely to occur when an individual anticipates that failure is likely to be aversive [7] and under those circumstances when the person delegates control to others, searches for approval, or fears disapproval from others. For example, regarding individual self-perceptions, De Castella, Byrne and Covington [7] in a study with more than two thousand high school students, noted that high fear of failure was positively related to helplessness and self-handicapping. Irrespective of the cause of the fear, students tend to desire to protect their self-esteem should their performance not reach expectations and the long-term effect of this type of fear is for the individual to experience diminished intrinsic motivation and feelings of uneasiness [8,9]. The most common characteristic associated with fear of failure does not involve negative personal feelings but rather the negative social stigma associated with the appearance of incompetence [10]. In addition, some previous work has indicated that teachers often avoid expressing the word ‘failure’ during their interactions with students [11]. Given the reciprocal and dynamic relationships among students and teachers [12–14], it is common for teachers to adjust their motivational style according to the personal characteristics of their students. Previous research has demonstrated that fear of failure in the educational context can contribute to the desire to avoid challenge and to seek to preserve self-esteem which, in turn, can diminish the personal and academic experience for the individual [15]. In the sport setting, these same concerns can contribute to an elevated anxiety response [16]. However, some recent research has revealed that failure can itself be beneficial by fostering new paths to divergent learning and even the experience of failure itself can be a springboard to a later experience of interest and enjoyment in completing a task [11]. So, reflective thinking after academic failure, encompassing beliefs, attribution, and affect, may potentially contribute to subsequent effort-making [17]. Similar research has identified potential value in failure as an opportunity for learning and perseverance [18], where the educator must be a guide to help the learners how to solve the problem for themselves [6]. In this sense, employing strategies that are known to reduce student feelings of stress, such as allowing them more time, providing more positive role modeling behavior, and allowing them to assist each other before providing instructor assistance are advised in accordance with previous research in the educational context [11]. In short, recent scientific knowledge provides evidence that constructive reflection after failure may provide students with strong motivation to persist and may provide them with an opportunity to build character in the process of achieving future academic success.

In addition to these considerations, student motivational characteristics may vary, at least in part, due to the interpersonal style of the instructor [19]. To the present time, limited knowledge has been gained about the effects of controlling instructional styles in comparison to autonomy-supportive instructional styles in contributing to student motivation and uneasiness in the academic setting [20], as the effects of controlling teaching styles have typically been examined with the sport context [21]. In this way, Self- Determination Theory, (SDT) [22,23] proposes that autonomy-supportive teachers promote feelings of autonomy by providing meaningful choices, acknowledging and accepting negative feelings, using non-controlling language and contributing to the development of autonomously-motivated students who have high levels of engagement and well-being [24,20]. Conversely, teachers with a controlling style can promote discomfort among their students. Thus, they can communicate to their students that only their perspective matters, even to the point of using threats in order to induce them to think, feel and act in accordance with the preferred style of the teacher [25,26]. In order for teachers to successfully facilitate well-being in students, it is crucial that the student perceives that the teaching environment is motivationally supportive [27].

Limited research suggests that fear of failure is the consequence of student perceptions of incompetence and the desire for task avoidance in response to perceptions of teacher control [25]. As Bartholomew and colleagues [28] have noted, feelings of uneasiness are typically the result of the frustration of students’ basic psychological needs and so to understand avenues of influence for understanding the effects of teaching styles upon motivation it is essential to achieve the goal of optimizing learning outcomes [29]. In this line of study, Conroy et al. [4] demonstrated that perceptions of failure may depend more heavily upon the extent to which participants’ basic psychological needs have been satisfied rather than upon their actual competence levels. Moreno and Silveira [30] found that positively-oriented climates are inversely associated with students’ fear of failure and that students’ intrinsic motivation was predicted by positive feedback and low fear of failure.

A current avenue of study that stems from Self-Determination Theory [22,23] involves the desire to understand adaptive and maladaptive styles of students in relation to controlling teaching styles. Until the present time, the majority of the research conducted on SDT has focused on a variable-centered approach, with the aim of explaining relationships and effects between variables of interest placing the teacher as a social antecedent contributing to a series of cognitive, emotional, and behavioral consequences in students. Although relevant findings have been obtained in this line of research, this approach has not been able to identify unique subgroups of students within the larger population with regard to their students’ responses to teaching practices. Thus, a fundamental purpose of this study was thus to examine the relationship between teaching styles and fear of failure in the physical education context. It was proposed that an autonomy-supportive style perceived would result from lower levels of fear of failure among students than would a controlling style. This expectation was based on knowledge that a controlling style tends to contribute to perceptions of threat and uneasiness on behalf of students [25,26,31]. Although there is a body of knowledge on the influence of instructor behavior on student cognitive, behavioral and emotional outcomes, relatively few studies have examined how individual student characteristics are affected by the student’s perception of teachers’ motivational style [21]. Initial evidence for such an argument comes from recent scientific literature with PE students in which perceptions of controlling teaching were associated with maladaptative outcomes like fear to failure [32], as well as helplessness beliefs and lesson avoidance behaviors [33].

## Methods

### Participants

The sample was randomly selected and was comprised of 562 students (236 males and 326 females) who were completing their third or fourth year of middle school or the first year of their high school education. A total of 20 physical education classes and their teachers participated (16 teachers directed the groups, since four of them taught in two classes simultaneously), with an average of 28.1 students per class. Within their regular academic calendar, physical education was a required course, and students received two hours a week of classes in two sessions for one hour. The students had a mean age of 15.43 years (*SD* = 1.09 yrs.) and represented three public educational institutions in Spain.

### Measures

#### Fear of failure

The Performance Failure Appraisal Instrument as developed by Conroy, Willow and Metzler [5] and validated for use in the Spanish language and cultural context by Moreno and Conte [34] was used in the present study. The instrument is comprised of 25 items and students respond to the common stem question of, “In performing my sport…” The Spanish language version of the instrument contains five subscales that are grouped along five categories, two of which are comprised of five items; two are comprised of four items; and one is comprised of seven items. Subscales are labeled, *Fear of Experiencing Shame and Embarrassment* (e.g., “When I am not succeeding, I am less valuable than when I succeed”); *Fear of Devaluing One’s Self-Estimate* (e.g., “When I am failing, it is often because I am not smart enough to perform successfully”); *Fear of Having An Uncertain Future* (e.g., “When I am failing, my future seems uncertain”); *Fear of Important Others Losing Interest* (e.g., “When I am not succeeding, people are less interested in me”); and *Fear of Upsetting Important Others* (e.g., “When I am failing, it upsets important others”). The response format is structured along a 5-choice Likert type format ranging from “1” (“Do not believe at all”) to “5” (“Believe 100% of the time”). The internal consistency for the individual subscales in the present study were .80, .81, .69, .77 and .75, respectively.

#### Autonomy support

Participants completed the Autonomy Support Scale [35] measuring the individual’s perception of their teacher’s use of autonomy supportive behaviors. All 12-items (e.g., “*Throughout the class invites us to make proposals and values our ideas and suggestions”*) were preceded by the sentence “*My teacher…*” and participants responded to each item using a 5-point scale anchored from 1 (totally disagree) to 5 (*totally agree*). A Cronbach alpha value of .81 was found as an index for the internal consistency of this scale.

#### Controlling teaching style

The Controlling Behavior Scale [36] was used to assess students’ perception of their teacher’s level of controlling behaviors. This scale is composed of 12 items (item example: “*My teacher shouts in front of the others to behave in a certain way*”), to which participants responded on a 7-point bipolar scale anchored from 1 (totally disagree) to 7 (totally agree). A Cronbach alpha value of .80 was found for the internal reliability of this scale.

### Study procedure and design

The directors of the educational centers that would be potentially involved in this study were contacted for the purpose of soliciting their interest and cooperation. Approval was first obtained through the Research Ethics Committee (Oficina de Investigación Responsable, OIR) at the principal investigator’s university and permission was subsequently obtained from the parents and teachers who were involved before student assent was requested (protocol number DPS.JMM.01.14). The questionnaires were administered in classes or in practice sessions and the lead investigator supervised the administration of all questionnaires and assisted individuals who had any questions. The participants completed their questionnaires in an anonymous fashion and typically needed about fifteen minutes to complete the task.

### Data analysis

Descriptive statistics, including means and standard deviations, were calculated for all of the variables in the study. Cronbach alpha coefficients were computed to assess the internal consistency of each of the instruments and bivariate correlations were also computed among the variables. A cluster analysis was carried out in order to identify the fear of failure profiles. The purpose of this analysis was to identify homogeneous groups or clusters based on their common characteristics. The motivational characteristics associated with each profile were examined through analysis of variance procedures. SPSS 25.0 was used as the statistical package throughout the analyses.

## Results

### Descriptive analyses

Descriptive analyses were conducted and examined for each of the variables (Table 1). With regard to fear of failure, mean values were found to be highest for the subscales related to fear of experiencing failure and fear of personal devaluation. Negative correlations were found between autonomy support and personal devaluation as well as between autonomy support and fear of losing the interest of essential others. The controlling style correlated in a positive direction with each of the fear of failure dimensions. Autonomy support and controlling styles correlated significantly and negatively with each other as well. Each of the fear of failure dimensions correlated positively and significantly with each of the other dimensions.

**Table 1:** Means, standard deviations and correlations for all variables.

Variable	*M*	*SD*	Α	Range	1	2	3	4	5	6	7	8
1. Autonomy Support	3.29	.72	.81	1–5	-	.46**	-.06	-.09*	-.01	-.08*	-.07	-.07
2. Controlling Style	2.56	.66	.80	1–5	-	-	.10*	.16**	.10*	.16**	.13**	.13**
3. Fear of experiencing embarrasment	2.40	.97	.80	1–5	-	-	-	.69**	.48**	.62**	.63**	.84**
4. Fear of devaluing one`s self-estimate	2.21	.82	.81	1–5	-	-	-	-	.55**	.46**	.55**	.72**
5. Fear of having a uncertain future	1.85	.95	.69	1–5	-	-	-	-	-	.42**	.61**	.68**
6. Fear of losing the interest of important others	1.73	.81	.77	1–5	-	-	-	-	-	-	.63**	.73**
7. Fear of upsetting important others	1.83	.77	.75	1–5	-	-	-	-	-	-	-	.77**
8. General Fear	2.02	.83	.83	1–5	-	-	-	-	-	-	-	-

*p* < .05*; *p* < .01**.

### Cluster analysis

**Fig 1:**
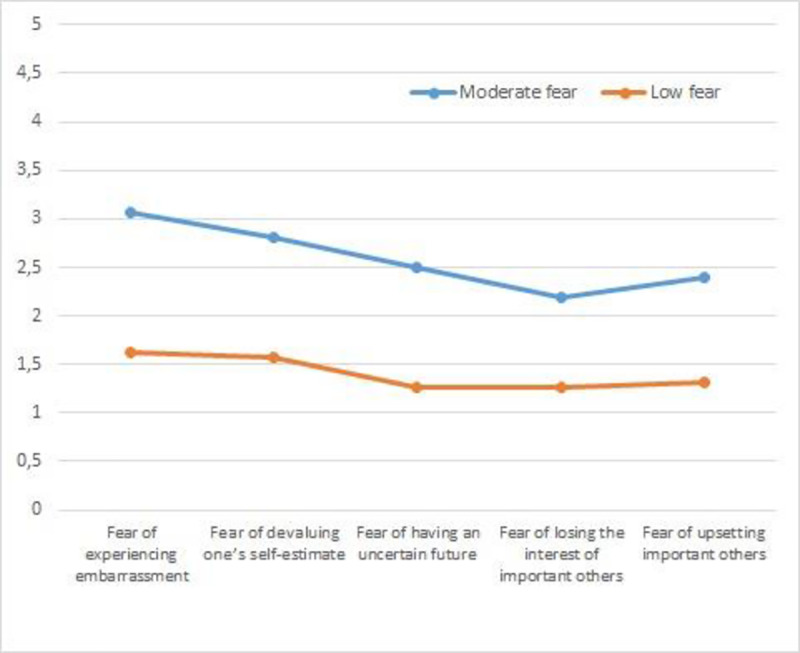
Profiles fear of failure in sample 1.

**Fig 2:**
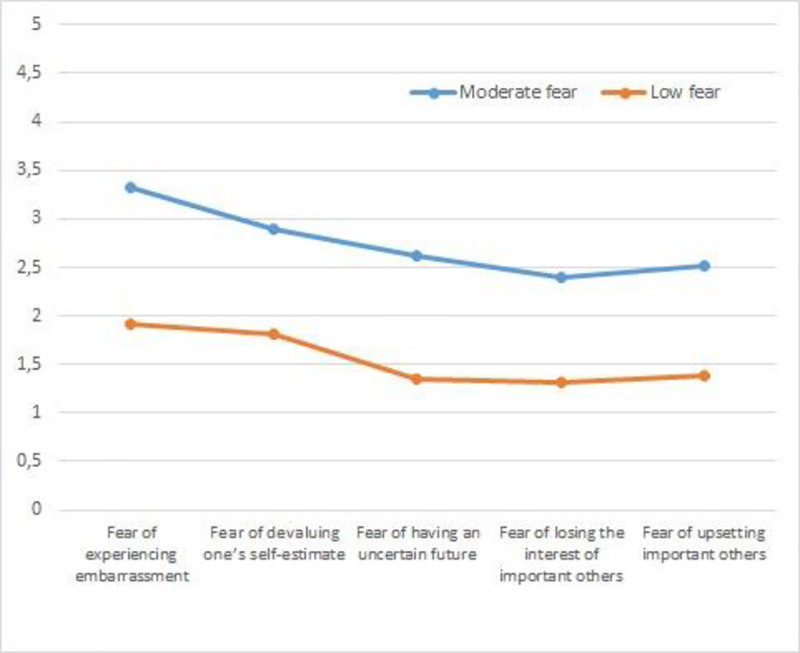
Profiles fear of failure in sample 2.

The cluster analysis was conducted in relation to the steps proposed by Hair, Anderson, Tatham, and Black [37]. The total sample was separated into two subsamples through a random process. With Sample 1 (*n* = 292) an agglomerative hierarchical analysis was conducted using Ward’s method. To determine the adequacy of the clusters that were identified, incremental agglomeration coefficients were examined. In accordance with the approach recommended by Norusis [38], smaller coefficients reflect greater homogeneity among members of a given cluster whereas larger coefficients indicate larger differences among cluster members. The dendogram that was obtained provided evidence for the presence of two clusters of similar sizes (*n* = 129 & *n* = 124). In Fig 1, there is a representation of these cluster profiles which are labeled “Moderate Fear” (Cluster 1) and “Low Fear” (Cluster 2). On a scale of 1–5, considering 3.0 as the midpoint value for the scale, we established that values between 2.0 and 3.0, would be labeled as “moderate” and that values below 2.0, would be labeled “low” on the corresponding variables. Significant differences between the clusters were revealed by subsequent multivariate analysis (Wilks Λ = .31, *F* (5, 247) = 108.98, *p* < .001, η^2^ = .68). Univariate analyses revealed significant differences for the two clusters in their cluster means on fear of experiencing shame and embarrassment, *F* (5, 247) = 311.24, *p* < .001, η^2^ = .55; fear of devaluing one’s self-estimate, *F* (5, 247) = 268.26, *p* < .001, η^2^ = .51; fear of having an uncertain future, *F* (5,247) = 200.96, *p* < .001, η^2^ = .44; fear of important others losing interest, *F* (5, 247) = 124.20, *p* < .001, η^2^ = .33; and fear of upsetting important others, *F* (5,247) = 207.16, *p* < .001, η^2^ = .45. To identify the clusters in Sample 2 (Fig 2), a K Means test was conducted and two profiles emerged that consisted of 114 and 195 students (Table 2).

**Table 2:** Standardized values, means and standard deviations of the variables in each cluster for Sample 1, 2 and total.

	Sample 1				Sample 2				Total Sample			
Variables	Cluster 1		Cluster 2		Cluster 1		Cluster 2		Cluster 1		Cluster 2	
	(*n* = 129)		(*n* = 124)		(*n* = 114)		(*n* = 195)		(*n* = 292)		(*n* = 270)	
	Moderate Fear		Low Fear		Moderate Fear		Low Fear		Moderate Fear		Low Fear	
	*M*	*SD*	*M*	*SD*	*M*	*SD*	*M*	*SD*	*M*	*SD*	*M*	*SD*
1. Fear of experiencing embarrasment	3.06	.78	1.62	.47	3.33	.74	1.91	.67	3.09	.77	1.65	.49
2. Fear of devaluing one`s self-estimate	2.80	.58	1.58	.60	2.90	.57	1.82	.65	2.75	.61	1.64	.62
3. Fear of having an uncertain future	2.50	.90	1.26	.38	2.62	1.02	1.35	.51	2.42	.97	1.24	.37
4. Fear of losing the interest of important others	2.19	.82	1.27	.40	2.40	.91	1.32	.43	2.17	.87	1.26	.37
5. Fear of upsetting important others	2.40	.73	1.32	.41	2.52	.70	1.38	.35	2.31	.74	1.32	.37

**Fig 3:**
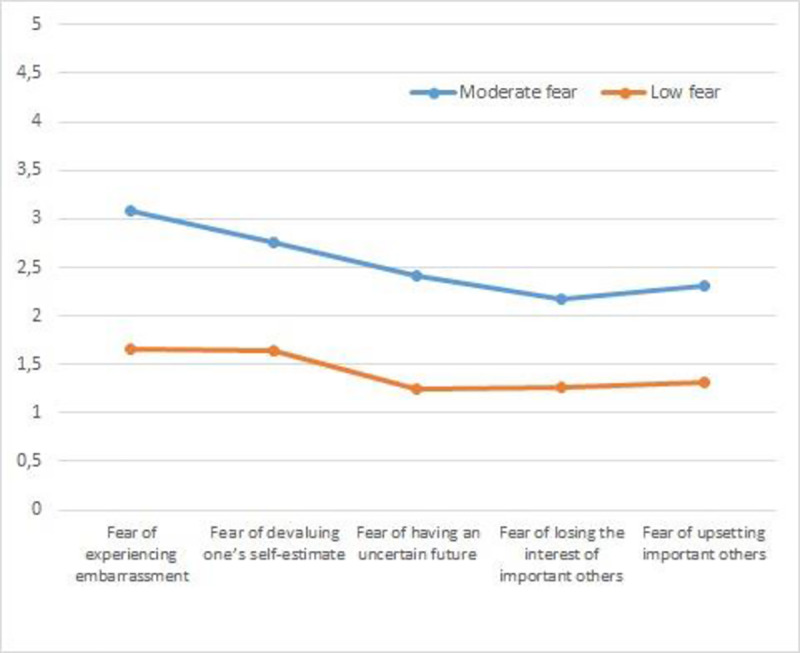
Profiles fear of failure in total sample.

Cluster 1 was labeled “Moderate Fear” with scores on the fear scale that were near the scale’s midpoint. Cluster 2 was labeled the “Low Fear” group as mean scores for this cluster were generally low on all of the variables included. Multivariate analysis revealed significant differences between the clusters, Wilks Λ = .28, *F* (5, 303) = 152.32, *p* < .001, η^2^ = .71) and follow-up univariate analysis revealed significant differences in fear of experiencing shame and embarrassment, *F* (5, 503) = 298.77, *p* < .001, η^2^ = .49; fear of devaluing one’s self-estimate, *F* (5, 503) = 216.99 *p* < .001, η^2^ = .41; fear of having an uncertain future, *F* (5, 503) = 211.33, *p* < .001, η^2^ = .40; fear of important others losing interest, *F* (5,503) = 195.87, *p* < .001, η^2^ = .39; and fear of upsetting important others, *F* (5,503) = 362.18 *p* < .001, η^2^ = .54. An agglomerative hierarchical analysis using Ward’s method was also performed with groups of 292 and 272 students, respectively (Fig 3). The results were similar to those obtained in the previous cluster analysis with a significant overall difference across the dimensions, Wilks Λ = .33, *F* (5, 556) = 218.76, *p* < .001, η^2^ = .66. Univariate differences were also found for fear of experiencing shame and embarrassment, *F* (1, 338) = 679.85, *p* < .001, η^2^ = .54; fear of devaluing one’s self estimate, *F* (1, 338) = 457.34, *p* < .001, η^2^ = .45; fear of having an uncertain future, *F* (1, 338) = 346.40 *p* < .001, η^2^ = .38; fear of important others losing interest, *F* (1, 338) = 248.20, *p* < .001, η^2^ = .30; and fear of upsetting important others *F* (1, 338) = 382.13, *p* < .001, η^2^ = .40.

### Differential analysis

To test for possible differences between the two cluster profiles on fear of failure and perceptions of teaching styles, a differential analysis was conducted with the clusters serving as the independent variable and the teaching style as the dependent variable. Significant differences were identified, Wilks Λ = .97, *F* (2, 559) = 7.33, *p* < .001, η^2^ = .03. The results revealed significant differences in teaching style in relation to autonomy support, *F* (1, 559) = 5.20, *p* < .05, η^2^ = .01, with higher levels of autonomy support present in the “low fear” cluster (*M* = 3.37, *SD* = .73) in comparison to the “moderate fear” cluster (*M* = 3.37, *SD* = .73). Significant differences were also present in relation to perceptions of a controlling teaching style, *F* (1, 559) = 14.31, *p* < .001, η^2^ = .02, with higher levels in the “Moderate Fear” cluster (*M* = 2.67, *SD* = .69) than in the “Low Fear” cluster (*M* = 2.45, *SD* = .61. These results are presented in Table 3.

**Table 3:** Univariate analysis of teachers’ interpersonal style (autonomy supportive and controlling style) with the total sample.

Variables	Cluster 1		Cluster 2			
	(*n* = 292)		(*n* = 270)			
	*M*	*SD*	*M*	*SD*	*F*	*η*^*2*^
Autonomy Support	3.23	.71	3.37	.73	5.20*	.01
Controlling Style	2.67	.69	2.45	.61	14.31**	.02

*p* < .05*; *p* < .01**.

## Discussion

Based on the postulates of SDT [20,22,23], this study examined the influence of the learning context on student psychosocial outcomes. Given the potential influence of teaching styles on student outcomes, the propositions contained in SDT are very relevant to the consideration of the effects of instructors’ need supportive and controlling teaching styles on students fear of failure in physical education classes. There have been few studies conducted to date that have examined the motivational style of teachers in relation to students’ fear of failure in physical education [3,4] and the purpose of this study was to assess these relationships through cluster analysis within the physical education context. The proposed hypothesis was supported in that perceived autonomy-supportive styles were inversely, and significantly, correlated with fear of failure whereas a controlling teaching style was positively and significantly correlated with fear of failure. Based on the previous scientific evidence on the bidirectional, reciprocal and dynamic influence of various psychosocial variables related to motivation in educational contexts, it is useful to contemplate the role of student characteristics (antecedents), as well as outcomes (consequences), in interaction with teacher behavior [11–14]. In this regard, teachers respond to student characteristics to some extent and can adapt their pedagogical practices accordingly.

The two cluster profiles that emerged indicated that students’ fear of failure extended across the various dimensions of fear that were assessed and were associated with the controlling teaching style. These results provide additional support for research that has come out of the SDT knowledge base and which has demonstrated that a positive class climate, in which the instructor demonstrates warmth and concern through an interactive style that provides support for student autonomy, results in favorable outcomes for students [39–41]. These results are of great value in the design of learning environments that are intended to minimize maladaptive learning outcomes. Some previous research [11] has highlighted the potential benefits of failure in the educational context as a stimulus to subsequent learning, particularly when the instructor can demonstrate how to respond to failure and how to initiate new approaches to learning. Research [42–44] indicates that certain learning climates can foster avoidance behaviors and contribute to fear of committing mistakes. In their quasi-experimental study, Silveira and Moreno-Murcia [3] found that satisfaction of students’ competence needs and the provision of positive feedback were associated with reduced fear of failure for students in the physical education context. Those students with greater intrinsic motivation would, logically, be those students with the least fear of failure. The findings from this study conform to previous research [45–48] in that the nature of instructors’ interpersonal style can have a positive or negative influence on students and interventions to enhance teachers’ interpersonal approaches may, therefore, prove beneficial to motivational and learning outcomes [49,50].

It is important to highlight the exploratory nature of this study as well as limitations associated with cluster analysis, which is essentially a correlational analysis in which cause-and-effect cannot be inferred. Further research will be needed to determine the extent to which our findings are replicated. It would also be interesting to include both the satisfaction and frustration of basic psychological needs as variables of study, and to assess their relationship with intrinsic motivation and instructors’ teaching styles. Future research would also benefit from research that uses a true experimental design and includes participants who vary in relation to gender and type of sport or physical activity involvement.

This study highlighted the potential relationship between teachers’ interpersonal styles and fear of failure in physical education students. This knowledge can be useful in the design of instructional programs that focus on expanding the evidence already provided by existing research [11] about the specific strategies that teachers could adopt to minimize fear of failure in their students. Autonomy-supportive behaviors on behalf of the teacher are one such strategy to reduce student fear of failure and these practices can contribute to more desirable learning climates for students.

## References

[1] Deng X., Sang B., Ku Y. & Sai L. Age-related differences in the late positive potential during emotion regulation between adolescents and adults. *Scientific Report*. 9, 10.1038/s41598-019-42139-4 (2019). PMC6451025 30952904

[2] Suárez J. M., Fernández A. P., & Anaya D. Un modelo sobre la determinación motivacional del aprendizaje autorregulado. [A model of self-regulated and self-determined motivation]. *Revista de Educación*, 338, 295–306.(2005).

[3] Silveira Y. & Moreno-Murcia J. A. Miedo a equivocarse y motivación autodeterminada en estudiantes adolescentes. [Fear of failure and self-determined motivation in adolescent students]. *Cuadernos de Psicología del Deporte*, 15 *(*3*)*, 65–74. (2015)

[4] Conroy D. E., Poczwardowski A., & Henschen K. P. (2001). Evaluative criteria and consequences associated with failure and success for elite athletes and performing artist. *Journal of Applied Sport Psychology*, 13, 300–322.

[5] Conroy D. E., Willow J. P., & Metzler J. N. (2002). Multidimensional fear of failure measurement: The performance-failure appraisal. *Journal of Applied Sport Psychology*, 14, 76–90.

[6] Manalo E., & Kapur M. (2018). The role of failure in promoting thinking skills and creativity: New findings and insights about how failure can be beneficial for learning. *Thinking Skills and Creativity*, 30, 10.1016/j.tsc.2018.06.001.

[7] De Castella K., Byrne D., & Covington M. (2013). Unmotivated or Motivated to Fail? A Cross-Cultural Study of Achievement Motivation, Fear of Failure, and Student Disengagement. *Journal of Educational Psychology*. 105 861–880. 10.1037/a0032464.

[8] Crocker J., Luhtanen R. K., Cooper M. L., & Bouvrette A. (2003). Contingencies of self-worth in college students: Theory and measurement. *Journal of Personality and Social Psychology*, 85, 894–908. 10.1037/0022-3514.85.5.894 14599252

[9] Zuckerman M., & Tsai F. F. (2005). Costs of self-handicapping. *Journal of Personality*, 73, 411–442. 10.1111/j.1467-6494.2005.00314.x 15745436

[10] Simmons S. A., Wiklund J., & Levie J. (2014). Stigma and business failure: Implications for entrepreneurs’ career choices. *Journal of Small Business Economics*, 42, 485–505.

[11] Lottero-Perdue P.S., & Parry E.A. (2017). Perspectives on failure in the classroom by elementary teachers new to teaching engineering. *Journal of Pre-College Engineering Education Research*,7(1) 47–67, 10.7771/2157-9288.1158

[12] Martínez D. (2007). Nuevas regulaciones, ¿nuevos sujetos? In Feldfeber M. & Oliveira D. (Comps.), *Políticas educativas y trabajo docente*, *nuevas regulaciones*, *¿nuevos sujetos*? (pp. 33–52). Buenos Aires: NOVEDUC.

[13] Escartí A., y Cervelló E. M. (1994). La motivación en el deporte In Balaguer I. (Ed.), Entrenamiento psicológico en deporte: Principios y aplicaciones (pp. 61–90). Valencia: Albatros Educación.

[14] Roberts G.C. (2001). Understanding the dynamics of motivation in physical activity: The influence of achievement goals and motivational processes In Roberts G. C. (Ed.), Advances in motivation in sport and exercise (pp. 1–50). Champaign: IL: Human Kinetics.

[15] Shim S., & Ryan A. (2005). Changes in self-efficacy, challenge avoidance, and intrinsic value in response to grades: The role of achievement goals. *The Journal of Experimental Education*, 73, 333–349.

[16] Correira M., Rosado A., & Serpa S. (2016). Fear of failure in sports: A Portuguese cross-cultural adaptation. *Motriz*, 22(4), 376–382.

[17] Tulis M., & Ainley M. (2011). Interests, enjoyment and pride after failure experiences? Predictors of students’ state-emotions after success and failure during learning in mathematics. *Educational Psychology*, 31 (7), 779–807.

[18] Maltese A., Simpson A., & Anderson A. (2017). Failing to learn: The impact to failures during making activities. *Thinking Skills and Creativity*, 30, 116–124. 10.1016/j.tsc.2018.01.003

[19] Oyama Y., Manalo E., & Nakatani Y. (2018). The Hemingway effect: How failing to finish a task can have a positive effect on motivation. *Thinking Skills and Creativity*, 30, 7–18. 10.1016/j.tsc.2018.01.001

[20] Reeve J. (2016). Autonomy-supportive teaching: What it is, how to do it In Liu W.C. et al (Eds.), *Building autonomous learning* (pp. 129–153). Singapore: Springer Science and Business Media.

[21] Bartholomew K., Ntoumanis N., Mouratidis A., Katartzi E., Thøgersen-Ntoumani C., & Vlachopoulos S. (2018). Beware of your teaching style: A school-year long investigation of controlling teaching and student motivational experiences. *Learning and Instruction*, 53, 50–63.

[22] Deci E. L., & Ryan R. M. (2000). The “what” and “why” of goal pursuits: Human needs and the self-determination of behavior. *Psychological Inquiry*, 11, 227e268. 10.1207/S15327965PLI1104_01.

[23] Ryan R. M., & Deci E. L. (2017). *Self-determination theory*: *Basic psychological needs in motivation*, *development*, *and wellness*. Nuevaew York: Guilford Publishing.

[24] Núñez J. L., Fernández C., León J., & Grijalvo F. (2015). The relationship between teachers’ autonomy support and students´ autonomy and vitality. *Teachers and Teaching*: *Theory and Practice*, 21, 191–202.

[25] De Meyer J., Soenens B., Aelterman N., De Bourdeaudhuij I., & Haerens L. (2016). The different faces of controlling teaching: implications of a distinction between externally and internally controlling teaching for students’ motivation in physical education. *Physical Education and Sport Pedagogy*, 21(6), 632–652. 10.1080/17408989.2015.1112777

[26] Reeve J., & Tseng C. (2011). Agency as a fourth aspect of students’ engagement during learning activities. *Fuel and Energy Abstracts*, 36 257–267. 10.1016/j.cedpsych.2011.05.002.

[27] Haerens L., Kirk D., & Cardon G. & Bourdeaudhuij I. (2011). Toward the development of a pedagogical model for health-based Physical Education. *Quest*, 63, 321–338. 10.1080/00336297.2011.10483684.

[28] Bartholomew K., Ntoumanis N., Ryan R., & Thøgersen-Ntoumani C. (2011). Psychological need thwarting in the sport context: Development and initial validation of a psychometric scale. *Journal of Sport & Exercise Psychology*, 33, 75, 102. 10.1123/jsep.33.1.75 21451172

[29] Jang H., Kim E. J., & Reeve J. (2016). Why students become more engaged or more disengaged during the semester: A self-determination theory dual-process model. *Learning and Instruction*, 43, 27–34. 10.1016/j.learninstruc.2016.01.002

[30] Moreno-Murcia J. A., & Silveira Y. (2013). Relación del feed-back positivo y el miedo a fallar sobre la motivación intrínseca. [Relationships between positive feedback and fear of failure on intrinsic motivation]. *REOP* 24 *(*2*)*, 8–23.

[31] Van den Berghe L., Soenens B., Vansteenkiste M., Aelterman N., Cardon G., Tallir I. B., et al (2013). Observed need-supportive and need-thwarting teaching behavior in physical education: Do teachers’ motivational orientations matter? *Psychology for Sport and Exercise*, 14, 650–661. 10.1016/j.psychsport.2013.04.006

[32] Ntoumanis N, Pensgaard AM, Martin C & Pipe K. (2004). An idiographic analysis of amotivation in compulsory school physical education. *Journal of Sport & Exercise Psychology*, 26(2), 197–214. 10.1123/jsep.26.2.197.

[33] Bartholomew K.J, Ntoumanis N., Mouratidis A., Katartzi E.S., Thøgersen-Ntoumani C., & Vlachopoulos S.P. (2018). Beware of your teaching style: A school-year long investigation of controlling teaching and student motivational experiences. 10.1016/j.learninstruc.2017.07.006

[34] Moreno-Murcia J. A., & Conte L. (2011). Predicción del miedo a equivocarse en jugadores de baloncesto a través del clima tarea de los iguales y la motivación intrínseca. [Prediction of fear of failure in basketball players engaged in a task climate based on intrinsic motivation]. *Revista Mexicana de Psicología*, 28(1), 43–52. http://www.redalyc.org/articulo.oa?id=243029630004

[35] Moreno-Murcia J. A., Huéscar E., Pintado R., & Marzo J. C. (2019). Diseño y validación de la escala de apoyo a la autonomía en Educación Superior: relación con la competencia laboral del discente [Design and validation of an autonomy support scale in Higher Education: Relationship with the student’s work competence]. *Revista Española de Orientación Psicopedagógica*, 30(1), 116–130.

[36] Moreno-Murcia J. A., Pintado R., Huéscar E., & Marzo J. C. (2018). Estilo interpersonal controlador y percepción de competencia en educación superior [Controlling interpersonal style and perception of competence in higher education]. *European Journal of Education and Psychology*, 11(1), 33–45.

[37] Hair J., Anderson R., Tatham R., & Black W. (1998). *Multivariate data analysis*. 5th Edition, Prentice Hall: New Jersey.

[38] Norusis M. J. (1992). *SPSS/PC+ Professional statistics*, *Version 5*.*0*. Chicago, IL: SPSS.

[39] Jang H., Reeve J., & Deci E. L. (2010). Engaging students in learning activities: It is not autonomy support or structure but autonomy support and structure. *Journal of Educational Psychology*, 102(3), 588–600. 10.1037/a0019682

[40] Reeve J. (2009). Why teachers adopt a controlling motivation style toward students and how they can become more autonomy supportive. *Educational Psychologist*, 44, 159–175. 10.1080/00461520903028990

[41] Reeve J. (2016). Autonomy-supportive teaching: What it is, how to do it In Wang J. C. K., Liu W. C. and Ryans R. M. (Eds.), Motivation in educational research: Translating theory into classroom practice (chap. 5). New York, NY: Springer.

[42] Bartholomew K. J., Ntoumanis N., Ryan R. M., Bosch J., and Thøgersen-Ntoumani C. (2011). Self-determination theory and diminished functioning: The role of interpersonal control and psychological need thwarting. *Personality and Social Psychology Bulletin*, 37, 1459e1473. 10.1177/0146167211413125 21700794

[43] Ng L., & Jenkins A. (2018). Motivated but no starting: How fear of failure impacts entrepreneurial intentions. *Small Enterprise Research*. 10.1080/13215906.2018.1480412

[44] Halliburton A. L., & Weiss M. R. (2002). Sources of competence information and perceived motivational climate among adolescent female gymnasts varying in skill level. *Journal of Sport & Exercise Psychology*, 24, 396–419.

[45] Ommundsen Y. (2001). Pupils’ affective responses in physical education classes: the association of implicit theories of the nature of ability and achievement goals. *European Physical Education Review*, 7, 219–242.

[46] Ruíz V., Gómez M., Granero A., & González J. (2017). Relación del clima motivacional y miedo al fallo en jugadores de alto rendimiento en balonmano. [Relationship between motivational climate and fear of failure in high-ability basketball players]. *Cuadernos de Psicología del Deporte*, 17(3), 55–64.

[47] Amoura C., Berjot S., Gillet N., Caruana S., Cohen J., & Finez L. (2015). Autonomy-supportive and controlling styles of teaching. Opposite or distinct teaching styles? *Swiss Journal of Psychology*, 74(3), 141–158.

[48] Cheon S. H., & Reeve J. (2014). A classroom-based intervention to help teachers decrease students' amotivation. *Contemporary Educational Psychology*, 40, 99–111.

[49] Cheon S. H., Reeve J., Lee Y., & Lee J. (2018). Why autonomy-supportive interventions work: Explaining the professional development of teachers’ motivating style. *Teaching and Teacher Education*, 69, 43–51.

[50] Cheon S. H., Reeve J., & Song Y.-G. (2016). A teacher-focused intervention to decrease PE students' amotivation by increasing need satisfaction and decreasing need frustration. *Journal of Sport and Exercise Psychology*, 38, 217–235. 10.1123/jsep.2015-0236 27385730

